# Bridging the evidence-to-practice gap: a stepped-wedge cluster randomized controlled trial evaluating practice facilitation as a strategy to accelerate translation of a multi-level adherence intervention into safety net practices

**DOI:** 10.1186/s43058-021-00111-2

**Published:** 2021-02-17

**Authors:** Antoinette Schoenthaler, Franzenith De La Calle, Amanda Soto, Derrel Barrett, Jocelyn Cruz, Leydi Payano, Marina Rosado, Samrachana Adhikari, Gbenga Ogedegbe, Milagros Rosal

**Affiliations:** 1 Department of Population Health, Center for Healthful Behavior Change, NYU Langone Health, 180 Madison Avenue, 752, New York, NY 10016 USA; 2 Preventive and Behavioral Medicine, Department of Medicine, University of Massachusetts Medical School, Worcester, MA USA

**Keywords:** Hypertension, Medication adherence, Latino persons, Practice facilitation, Primary care

## Abstract

**Background:**

Poor adherence to antihypertensive medications is a significant contributor to the racial gap in rates of blood pressure (BP) control among Latino adults, as compared to Black and White adults. While multi-level interventions (e.g., those aiming to influence practice, providers, and patients) have been efficacious in improving medication adherence in underserved patients with uncontrolled hypertension, the translation of these interventions into routine practice within “real world” safety-net primary care settings has been inadequate and slow. This study will fill this evidence-to-practice gap by evaluating the effectiveness of practice facilitation (PF) as a practical and tailored strategy for implementing Advancing Medication *Adherence for Latinos with Hypertension through a Team-based Care Approach* (ALTA), a multi-level approach to improving medication adherence and BP control in 10 safety-net practices in New York that serve Latino patients.

**Methods and design:**

We will conduct this study in two phases: (1) a *pre-implementation phase* where we will refine the PF strategy, informed by the Consolidated Framework for Implementation Research, to facilitate the implementation of ALTA into routine care at the practices; and (2) an *implementation phase* during which we will evaluate, in a stepped-wedge cluster randomized controlled trial, the effect of the PF strategy on ALTA implementation fidelity (primary outcome), as well as on clinical outcomes (secondary outcomes) at 12 months. Implementation fidelity will be assessed using a mixed methods approach based on the five core dimensions outlined by Proctor’s Implementation Outcomes Framework. Clinical outcome measures include BP control (defined as BP< 130/80 mmHg) and medication adherence (assessed using the proportion of days covered via pharmacy records).

**Discussion:**

The study protocol applies rigorous research methods to identify how implementation strategies such as PF may work to expedite the translation process for implementing evidence-based approaches into routine care at safety-net practices to improve health outcomes in Latino patients with hypertension, who suffer disproportionately from poor BP control. By examining the barriers and facilitators that affect implementation, this study will contribute knowledge that will increase the generalizability of its findings to other safety-net practices and guide effective scale-up across primary care practices nationally.

**Trial registration:**

ClinicalTrials.gov NCT03713515, date of registration: October 19, 2018.

Contributions to the literature

- The impact of practice facilitation on the implementation of evidence-based interventions to support hypertension management into safety-net practices remains largely untested.
- This study will identify *how* practice facilitation may work to expedite the translation process for implementing evidence-based multi-level interventions to improve health outcomes in safety-net settings.
- This study will also address questions about individual and practice-level factors likely to influence the implementation of such approaches. Published reports often omit important details about implementation fidelity (i.e., extent to which system changes are implemented), what kind of training or assistance practices need, and how these approaches fit within the practice culture.

## Background

Uncontrolled hypertension is one of the most prevalent primary care diagnoses and the single most important factor driving the high rates of cardiovascular-related mortality and health care expenditures in the United States (US) [1]. While recent national data show increasing trends in the awareness and treatment of hypertension among individuals from all racial/ethnic groups, disparities in blood pressure (BP) control persist. Among Latino adults, rates of BP control are lower than among non-Hispanic black and white adults (35%, 43%, and 48%, respectively) [2]. Rates of BP control are lowest among Latino persons of Central American, South American, Cuban, and Dominican descent [3]. These statistics may be explained by the disproportionately poorer adherence to antihypertensive medications among Latino patients compared to Black and White patients [4–7]. Data from NHANES III showed that hypertensive Latino adults reported the lowest levels of adherence to their antihypertensive medications (67%) compared to Black and White adults (77% for both racial groups) [8]. Similar findings were documented in the Health and Retirement Study, in which 53% of Latino participants with hypertension reported adhering to antihypertensive medications compared to 64% and 73% of White and Black older adults [4]. In a recent national survey, 32% of Latino adults reported being adherent to cardiovascular medications, compared to 39% and 50% of Black and White respondents [9].

Growing evidence shows that multi-level interventions (i.e., those that aim to influence the patient, healthcare providers, and clinic systems) can produce significant improvements in patients’ medication adherence and clinical outcomes [10, 11]. Our own work showed that a multi-level intervention that included an *office redesign component* built into the electronic health record (EHR; i.e., to identify, refer, coach, document, and track progress of patients with uncontrolled hypertension), *a provider support component* consisting of shifting self-management activities to clinic Medical Assistants (MAs), and a *patient engagement component* consisting of personalized health coaching by MAs significantly improved self-reported medication adherence (78% vs. 72%, *p* = 0.02) compared to enhanced usual care in a sample of Latino patients at a community health center [12]. However, evidence-based interventions often take up to 17 years to be translated into clinical practice [13], especially in the context of under-resourced “safety-net” practices, due to unique financial, administrative, and patient challenges that slow the implementation of new care innovations (e.g., patients have more complex health issues, limited reimbursement for self-management support) or can lead to significant disruptions in practice workflow (e.g., limited clinical capacity to meet demand for patient services) [14, 15].

Practice facilitation (PF) is one model for accelerating the implementation of evidence-based interventions into healthcare settings [16, 17]. Through PF, a specially trained individual (facilitator) works with healthcare teams to develop the skills to adapt and implement evidence-based approaches into routine care (e.g., by redesigning workflows to support team-based care) [18]. In contrast to single component (e.g., audit and feedback) and other multicomponent implementation strategies (e.g., Learning Collaborative), PF places emphasis on building organizational capacity to make evidence-based systems changes that are tailored to the practice context to achieve sustainable improvements in system, provider and patient-level outcomes [17]. One systematic review found that primary care practices that used PF were 2.76 times more likely to implement evidence-based strategies than usual care practices [19]. Several studies have found that the effects of PF were sustained for as long as 1 year post intervention [20–22]. Despite the growing evidence of effectiveness of PF in primary care, few studies have focused on understanding “*how to”* facilitate the implementation of evidence-based system-level approaches into routine practice within safety-net primary care settings [17]. Thus, the impact of PF on implementing evidence-based systems to support hypertension management in safety-net practices remains largely untested.

To address this gap in the evidence-base, this article describes the design and methods that will be used in the Advancing Medication *Adherence for Latinos with Hypertension through a Team-based Care Approach (ALTA)* trial, a multi-level approach to improving medication adherence and BP control. This cluster randomized trial will be conducted at ten safety-net practices in New York and will focus on Latino patients given the above-mentioned disparities in hypertension control in this patient population.

## Methods

### Study design

Using a mixed-methods approach, this study will be conducted in two phases: (1) a pre-implementation phase where we will refine the PF strategy, based on the Consolidated Framework for Implementation Research (CFIR) [23] to facilitate the implementation of ALTA; and (2) an implementation phase, guided by Proctor’s Implementation Outcomes Framework (IOF) [24, 25]. The primary aim of the implementation phase is to evaluate, in a stepped-wedge cluster randomized trial, the level of implementation fidelity (e.g., degree to which a program is delivered as intended) of ALTA at 10 safety-net primary care practices that serve Latino patients. The secondary aims are to compare the effects of PF versus usual care (UC) on BP control and medication adherence among Latino patients with uncontrolled hypertension who are non-adherent to their antihypertensive medications followed in the practices. We will also explore the potential moderating factors at the healthcare system-, provider/staff-, and patient-levels (e.g., organizational capacity to change, evidence-based practice attitudes) [26–30] that influence the level of implementation fidelity of ALTA at the practices.

**Fig. 1:**
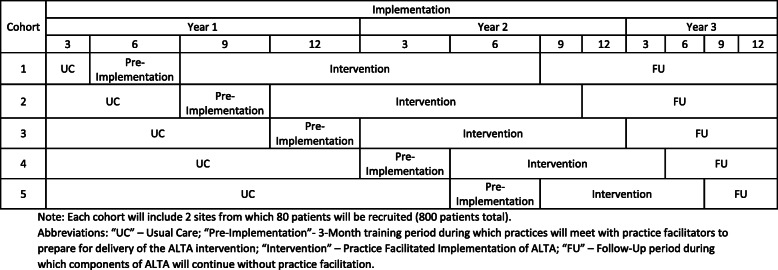
Stepped-wedge design of ALTA implementation

As shown in Fig. 1, each participating practice will start with the UC phase and are block-randomized to receive PF into four waves, with two sites per wave. The sequence of randomization for each block is generated by the study statistician and kept in sealed opaque envelopes away from the study sites in accordance with CONSORT guidelines.

The UC phase will be followed by the pre-implementation phase of 6 months, during which facilitators will conduct a practice evaluation (e.g., workflow analysis, environmental scan) based on CFIR, refine the PF strategies that will be used in the implementation phase, and train staff in the ALTA intervention. Following this period, sites will implement ALTA with the assistance of the PF for 12 months, with a 3-month follow-up period for outcome assessment. This study was approved by the Institutional Review Board of the New York University Langone Health (NYULH).

#### Study setting and participants

ALTA will be implemented as a standard of care at each of the 10 safety-net primary care practices participating in the trial. As such, study participants will include all practice providers/staff who will implement ALTA and patients seeking care at the practices. To address our secondary aims, we will focus our analysis on the subset of patients who identify as Latino adults (described below).

A Clinic Hypertension Committee consisting of the Site Directors, Director of the clinic Quality Improvement Committee, Nurse Managers, Patient Service Coordinators, Primary Care Providers, and Patient Advocates recruited across the practices will provide input on recruitment and retention strategies for the sites, providers/staff, and patients throughout the course of the trial.

### Recruitment

#### Primary care practices

Primary care safety-net practices will be selected and invited to participate with the assistance of Clinical Leadership who oversees the practices and outreach to the individual Site Directors. Practices will be eligible for participation if they (1) have an Epic EHR, (2) have used the EHR for at least 6 months, (3) have no immediate future plans to participate in a hypertension-related quality improvement initiative, (4) be willing to sign a Memorandum of Understanding and identify a practice champion to work with study staff on all aspects of the project implementation, (5) and be located in New York.

#### Practice staff and providers

We will recruit approximately ten clinical (e.g., primary care providers, nurses, medical assistants) and five non-clinical staff per practice to complete the study measures on the implementation of ALTA. A subset of these individuals will also be recruited to be part of the ALTA implementation team for their practice. Practice clinical and non-clinical staff will be recruited through study team attendance at the monthly staff meetings, email communications that describe the project including the overarching goals and what is expected of participants, and in-person meetings at the practices.

#### Patients

The analytic sample for the secondary aim will consist of patients who meet the following eligibility criteria: (a) identifies as Latino/a as documented in EHR codes; (b) be ≥ 18 years of age; (c) have uncontrolled hypertension defined using the 2017 guidelines for stage 1 [BP > 130–139 or 80–89 mmHg] and stage 2 [BP ≥ 140 or ≥ 90 mmHg] hypertension [31], as documented in the EHR on at least two visits in the past year; and (d) have been prescribed at least one antihypertensive medication and are non-adherent to their medication defined as adherence < 80% in the preceding 12 months, as determined by prescription orders obtained from the EHR. Patients will be excluded if they (a) currently participate in another hypertension study, (b) have significant psychiatric comorbidity or reports of substance abuse (as documented in the EHR), (c) are pregnant or planning to become pregnant within 12 months, or (d) plan to discontinue care at the practice within the next 12 months. As a multi-level study that will include the entire health system, all potentially eligible patients will be identified through a review of the EHR-embedded hypertension registry.

### Conceptual framework

The study uses two implementation science theoretical frameworks—CFIR and Proctor’s IOF. A strength of CFIR is the ability to tailor the framework to the specific intervention design and practice context being studied by exploring four major domains (intervention characteristics, external environment [outer setting], inner practice setting, and characteristics of individuals involved) that affect effective implementation of evidence-based practices [23, 32]. Proctor’s IOF guides in the evaluation of distinct implementation outcomes (implementation fidelity) that serve as indicators of implementation success of evidence-based practices [25]. Achieving a high level of implementation fidelity is necessary to attribute desired changes in clinical outcomes (e.g., improvements in BP control and medication adherence) to the effectiveness of the intervention and not to extraneous variables [33]. Diminished implementation fidelity is a primary reason why interventions conducted in controlled academic clinical settings do not yield similar results when translated to real-world primary care practices [33, 34].

**Fig. 2:**
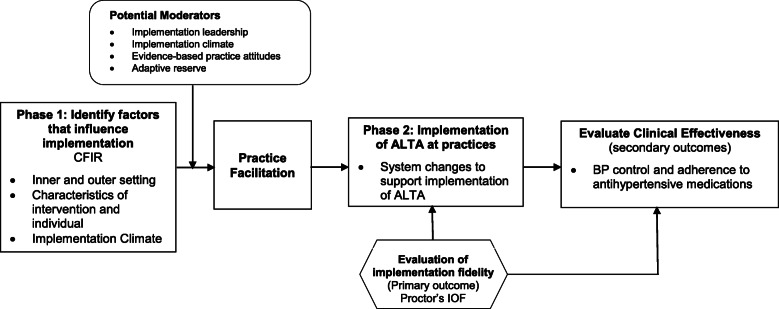
ALTA conceptual model

Our conceptual model (Fig. 2) illustrates the integration of our theoretical frameworks. The model hypothesizes that use of a tailored PF implementation strategy that targets the CFIR facilitators and barriers will result in a high level of implementation fidelity of ALTA (primary outcome) and that a higher proportion of patients will exhibit both improved BP control and medication adherence (secondary outcomes) during the PF implementation phase as compared to the UC phase. We will also explore the potential practice and individual-level moderating factors (e.g., organizational capacity to change, evidence-based practice attitudes) that influence the level of implementation fidelity of ALTA at the practices.

### Description of ALTA

ALTA is a multi-level approach to improving medication adherence and BP control in primary care practices. It is designed to build practice capacity to address the needs of hypertensive patients by utilizing a team-based approach to delivering hypertension care. ALTA includes five key components, which are conducted by existing members of the practice (Table 1): (1) *Identify* ALTA-eligible patients using EHR hypertension registries; (2) *Refer* ALTA patients to a clinic Health Coach using the EHR; (3) *Coach* ALTA patients using a structured counseling tool integrated into the EHR to identify patients’ treatment goals, asses their current level of BP control and medication adherence, identify barriers and facilitators to medication adherence, develop patient-centered adherence goals and action plans, and use a structured treatment algorithm to optimize patient antihypertensive medication regimen; (4) *Document* brief progress notes from the coaching session in the EHR to inform other team members of the patient’s adherence status, action plans, progress toward goals, and any changes to the antihypertensive medication regimen; and (5) *Monitor* and schedule follow-up sessions with ALTA patients in the EHR.

**Table 1:** ALTA components

ALTA components	ALTA activities
Identify	• EHR registry to identify Latino patients with uncontrolled Hypertension and are prescribed antihypertensive medications • Screen patients for medication non-adherence using a standardized adherence screening and document the result in the EHR
Refer	• Send EHR alert to clinic Health Coach (e.g., RN, MA) about patient’s BP level and adherence status
Coach	• Structured assessment of medication adherence • Use of motivational interviewing to identify facilitators/barriers to adherence and discuss personalized strategies to improve adherence behaviors • Use of structured medication titration algorithm to optimize patient treatment regimen • Use of teach back to ensure patient understanding of treatment plan • Co-creation of culturally-relevant goals and action plans
Document	• Structured documentation of coaching sessions in EHR to enable tracking patients’ progress over time • EHR Documentation of individualized treatment plan, medication adherence goals, and action plan to guide future sessions and share with primary care provider for clinic visits
Monitor	• Consistent monitoring of patient progress through completion of and tracking follow-up sessions with ALTA patients

*EHR* electronic health record, *MA* Medical Assistant, *ALTA* Advancing Medication Adherence for Latinos with Hypertension through a Team-based Care Approach

### Description of PF implementation strategy

#### Usual care (UC) phase

During the UC phase, patients at the sites will receive standard hypertension care delivered by their primary care providers.

#### Pre-implementation phase

Immediately following the UC phase and prior to the implementation of ALTA, all practices will participate in the pre-implementation phase for a period of 3 months. During the pre-implementation phase, facilitators will conduct a practice capacity assessment at each practice, including the identification of barriers and facilitators to the implementation of ALTA. This CFIR-guided assessment includes utilizing qualitative interviews, validated surveys, and environmental scans. In accordance with CFIR, the assessment will explore the (1) inner practice setting (e.g., leadership support, organizational capacity), (2) external environment (e.g., patient needs and resources, external resources and incentives), (3) staff characteristics (e.g., self-efficacy, knowledge, and beliefs about patient-centered counseling), and (4) intervention characteristics (e.g., complexity).

An environmental scan is a structured needs assessment that combines observational and survey data collection methodologies to develop a robust understanding of the internal conditions and external factors that affect implementation [35–39]. In this study, the environmental scan will serve the dual purpose of (1) developing a practice assessment to guide the refinement of the PF strategies that will be tested in the implementation phase and (2) giving the facilitators an opportunity to form relationships with practice staff and develop a shared understanding of project roles and responsibilities. To conduct the scan, facilitators will combine the CFIR observational tool with a structured workflow analysis and survey questions that assess staffs’ perceptions about the practice culture [40], beliefs about organizational change [28], and self-efficacy to conduct health coaching sessions [41]. Together, these data will be used to develop a robust understanding of the facilitators and barriers to implementation of ALTA at the practices.

Findings from the interviews and environmental scan will be synthesized into a report and shared with the Clinic Hypertension Committee. The Committee, in partnership with the study team, will use this report to tailor the PF strategy to the practice context to overcome identified challenges to implementation of ALTA into routine practice in the implementation phase. During this period, the study team will also hold a Kick-off Event at the practices that will include didactic and interactive sessions on topics such as the ALTA model components, best practices for implementing team-based models of care, defining roles and responsibilities in interdisciplinary care teams, developing effective interdisciplinary and patient-centered communication skills, and a discussion of the implementation and evaluation process. The study team will use strategies to maximize attendance of all clinical and non-clinical staff at the Kick-off Event. These include creating a shared vision for the project by working in partnership with practice leadership to develop workflows and strategies that complement and bolster current quality improvement initiatives for hypertension management; distributing incentives to practices and staff that recognize their role as “quality improvement champions” and show appreciation for their participation, and holding listening sessions with physicians and nurses to understand the unique needs and desires they have for improving hypertension in their patients.

#### Implementation phase

PF consists of building trusting relationships, fostering collaborative team-based problem solving, building effective communication, leveraging data and health information technology (HIT) to drive improvement, and establishing and sharing common goals between the facilitator and those engaged in making the change [42]. Through the PF strategy, facilitators are building practice’s skills and capacity to sustain workflows and care processes needed for ALTA and to continue to learn, monitor, and adapt it after the study has ended*.*

Our PF strategy is designed to stimulate specific, actionable steps that the practices can undertake to build a foundation that supports the integration of all five components of ALTA into primary care practices as routine care. This will combine one-on-one onsite tailored facilitation with opportunities for shared learning across practices through collaborative calls (Table 2).

**Table 2:** Practice facilitation (PF) strategies

ALTA component	ALTA PF strategy
Identify	• Assist clinic staff in identifying ALTA-eligible patient by using HYPERTENSION registries • Assist clinic staff in utilizing the HYPERTENSION registry to identify ALTA-eligible patients • Assist practices in optimizing the HYPERTENSION registry to simplify prioritization of HYPERTENSION patients that can benefit from health coaching
Refer	• Assist clinic staff in developing a referral workflow that supports referral to a health coach • Assist clinic staff in referring ALTA-eligible patients to a health coach
Coach	• Assist clinic staff in developing a health coaching session workflow that maximize the delivery of the intervention by using the ALTA structured tool to: o Reconcile HYPERTENSION medications and optimize treatment regimen using structured algorithm o Assess patient’s treatment goals o Assess medication adherence o Identify adherence barriers and facilitators o Develop goal/action plan for improved adherence • Assist clinic staff in using patient-centered communication
Document	• Assist clinic staff in documenting short progress notes with the patient’s HYPERTENSION treatment regimen, adherence level, and adherence goal/action plan in the EHR to inform other team members of the patient’s status
Monitor	• Assist clinic staff in identifying opportunities for follow-up • Assist clinic staff in scheduling and using calls, text or portal messages to monitor and contact patients in need of follow-up
**ALTA quality improvement component**	**ALTA PF strategy**
Dashboards/reporting	• Assist clinic staff in allocating resources for quality improvement activities and developing a reporting process to assess fidelity • Assist clinic staff in assessing quality improvement activities with HYPERTENSION management performance dashboards
Establish operational systems	• Assist clinic staff in optimizing EHR functionality to support adherence to ALTA interventions • Assist clinic staff in identifying a workable HYPERTENSION workflow that includes the ALTA key drivers • Assist staff to identify and resolve barriers to implementation fidelity

The rationale for onsite visits is informed by expert consensus that in-person visits are essential to establish and maintain an effective working relationship between sites and PFs [16]. PFs will coordinate meetings with practices, assist them in setting performance goals, train clinic staff in the key components of ALTA as well as in quality improvement strategies for practice redesign (e.g., workflow mapping), strategize on implementing ALTA-related practice changes, assist teams in testing workflow changes using the Plan-Do-Study-Act cycle, and share best practices for implementing ALTA within and across practices [16–18, 43]. A quality improvement and implementation manual informed by these tasks will be developed by the study team to drive the delivery of the PF strategy and implementation of ALTA.

Each PF will be responsible for five practices and will conduct a minimum of 13 site visits over the 12-month implementation period. These design decisions (number of site visits, ratio of facilitators to practices) are based on a systematic review of PF research and our experience implementing similar studies [16, 43, 44].

#### Practice facilitator (PF) training and supervision

All PFs will have clinical and/or managerial experience in primary care settings and will have received training through the HealthTeamWorks Quality Improvement Training program, which is based upon AHRQ’s Practice Facilitation Handbook [18]. The training covers four core competencies of PF: data use to drive improvement, interpersonal skills, HIT optimization, quality improvement, and change management methods [45]. In the first year of the project, we will add curriculum components to meet the specific goals of this study (e.g., education on the updated hypertension guidelines) as well as training tailored to the specific ALTA components, including system changes that we propose to implement, and data extraction from EHRs. The training will combine didactic sessions and case-based learning and will include booster sessions. A PF Supervisor will hold weekly meetings to help PFs (1) develop and maintain relationships, (2) maintain effective boundaries to allow the practice to build capacity, (3) acquire the content knowledge needed for the PF strategy, (4) monitor their progress through the implementation of change concepts, (5) troubleshoot problems, and (6) review their activity reports.

### Evaluation

We will use a mixed methods approach to collect process and clinical-related outcomes at the patient, staff, and practice-levels to evaluate the implementation of ALTA into routine care within the safety-net practices. The primary outcome is a degree of implementation fidelity measured at the practice and staff-levels. Secondary outcomes at the patient-level will include BP control and medication adherence. Implementation fidelity will be assessed on an ongoing basis throughout the implementation phase. Study assessments for the secondary clinical outcomes will be completed at baseline visit and 12 months.

#### Primary outcome measure

##### Implementation fidelity

**Fig. 3:**
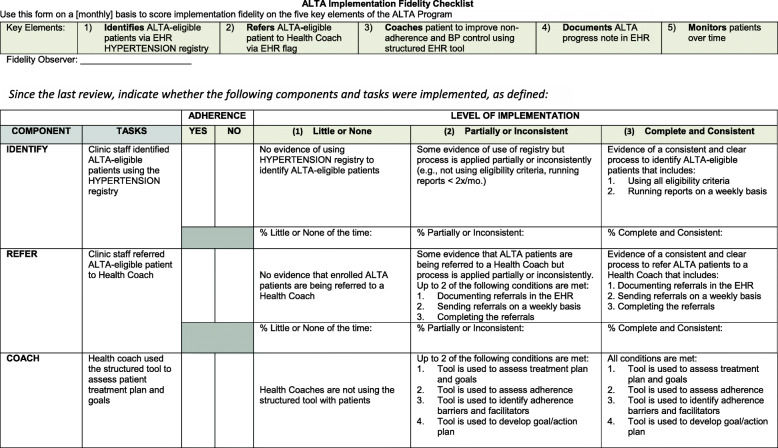
Excerpt of structured tool to assess implementation fidelity

Fidelity to ALTA will be defined by five core dimensions, as defined by Proctor’s IOF [25, 46]: (1) adherence to the program protocol, (2) dose of the program delivered, (3) quality of program delivery, (4) participant responsiveness, and (5) program differentiation. Each of these dimensions will be assessed using a structured tool developed for this study (Fig. 3) and defined in the following manner:

*Implementation adherence* will be evaluated as the degree to which the components of ALTA (e.g., identify, refer, coach, document, monitor) were implemented as intended, using data from checklists completed by the facilitators as well as extracted from the EHR. Each intervention component will be rated on a 3-point scale: 1 = the component was not implemented, as per protocol, 2 = the component was partially implemented, and 3 = the component was fully implemented and/or modified with permission, as per protocol. PFs will also complete bimonthly narrative reports that summarize all adaptations made to the components and what did/did not work for each practice context.

*Implementation dose* will be evaluated as the extent to which patients were exposed to ALTA. We will collect data on utilization patterns of the different ALTA components including the mean number of ALTA-eligible patients identified in the registry and referred to a Health Coach, health coaching sessions completed with ALTA patients, entries in the EHR-embedded coaching script, progress notes documented in the EHR and shared with the care team, and follow-up sessions that are scheduled and/or completed. System files will be extracted quarterly and will contain date and time stamps as well as user logins for the tools used.

*Implementation quality* of each ALTA component will be evaluated as the quality and content of data entry in the hypertension registry, EHR-embedded coaching script, progress notes, and follow-up scheduling. PFs will rate the completeness of this form using a 3-point rating scale (1, poor; 2, adequate; 3, high). A random 10% sample of counseling sessions by the clinic Health Coaches will also be audiotaped and evaluated using the Health Coaching Evaluation Checklist, as we have done in our previous trials [47]. A fidelity score will be calculated as the percentage of topics completed and how well they were delivered (1, poor skill performance; 2, adequate skill performance; 3, exemplary skill performance).

*Participant responsiveness* will be evaluated as patient and practice staff/provider satisfaction with ALTA and acceptability of practice changes with validated measures (i.e., ECHO survey for patients and a modified survey for staff/providers) [48, 49]. In addition, qualitative interviews will be conducted with a random sample of clinic staff (*n* = 30) and patients (*n* = 30) across the practices to assess satisfaction with the intervention.

*Program differentiation* will be evaluated as the unique features of ALTA that are distinguishable from other programs at the sites. Throughout the study, facilitators will catalog all initiatives that are occurring at the practices (as observed during site visits and/or reported by the practice leadership). This will be used to quantify the degree of overlap between the ALTA components and other quality improvement initiatives at the sites (1, no overlap; 2, some overlap; 3, significant overlap) as well as isolate the unique features of ALTA that distinguish it from those initiatives.

#### Secondary outcome measures

*BP control* will be defined as SBP < 130 and DBP < 90 mmHg [31]. BP control will be assessed using the mean of the last two measurements recorded in the EHR during each of the pre-intervention and post-intervention study periods (baseline and 12 months). BP readings will be extracted electronically from patients’ EHR using the centralized Clinical Research Data Management Core (DataCore) group at NYULH. We will use the last two recorded measures to reduce variability associated with multiple BP measurements being taken by different clinic staff. We will also explore the effect of PF on continuous BP reduction, defined as the mean change in systolic and diastolic BP based on the mean of the last two recorded BP readings in the EHR during the pre- and post-implementation periods.

*Medication adherence* will be assessed via pharmacy refills obtained from prescription orders in the clinic EHR using the proportion of days covered (PDC) metric. The PDC is calculated as the total number of days covered by the medication divided by the number of days between the first fill of the medication in the study period and the end of the study period [50]. The PDC accounts for non-persistence with medications by tracking medications during the study period despite early discontinuation. We will count the days the patient was covered by each of their prescribed antihypertensive medications based on the prescription fill date and days of supply [51]. If prescription refills for the same medication overlap, then we will adjust the prescription start date to be the day after the previous fill has ended. Patients will be considered adherent if they fill ≥ 80% of their antihypertensive medications over the 12 months. In addition, we will assess the extent of self-reported medication non-adherence and reasons for non-adherence using a survey developed by Voils and colleagues [52, 53].

#### Moderator measures

We will evaluate the potential moderating roles of practice- and provider/staff-level factors on implementation fidelity of ALTA at the practices, administering measures at baseline. All measures will be administered to clinical and nonclinical staff and administrative leadership at the practices. Staff will be invited to complete the measures via a broadcast email, which will contain a link to a secure website (i.e., RedCap) that includes the consent form and surveys. To maximize participation, staff will receive a $25 gift card for completing the measures.

*Adaptive reserve* is defined as a practice’s ability to make and sustain change and includes measures of facilitative leadership, relationship infrastructure, and culture of learning. Adaptive reserve will be assessed with the 23-item scale developed for the National Demonstration Project Model of the Patient-Centered Medical Home (*α* = 0.86) [54].

*Implementation climate* will be assessed with the 18-item Implementation Climate Scale (ICS) that measures shared perceptions of the policies, practices, procedures, and behaviors that are expected, supported, and rewarded to facilitate effective evidence-based practice (EBP) implementation (*α* = 0.91) [29].

*Implementation leadership* will be assessed with the 12-item Implementation Leadership Scale (ILS), which has excellent reliability, and convergent and discriminant validity(*α* = 0.98) [27]. The ILS is comprised of four subscales—proactive leadership (*α* = 0.95), knowledgeable leadership (*α* = 0.96), supportive leadership (*α* = 0.95), and perseverant leadership (*α* = 0.96).

*Evidence-based practice* (EBP) attitude scale is a 15-item measure with four subscales that assess attitudes toward the implementation of EBP as a function of the perceived appeal of EBP, requirements to use EBP, provider openness, and perceived divergence between EBP and usual care. Total scores (*α* = 0.76) represent global attitudes toward adoption of EBP [26].

#### Covariate measures

##### Practice and provider/staff characteristics

At baseline, we will collect data on the number of full-time equivalent staff, insurance payer mix, clinic volume, practice structure, staff composition (e.g., number of providers, nurses, MAs), use of HIT, current quality improvement initiatives, and other current or planned programs at each practice.

##### Patient sociodemographic and clinical characteristics

At baseline, we will extract data from the clinic EHR on patient race and ethnicity, primary language spoken, gender, insurance status, prescribed antihypertensive medications, changes in antihypertensive medications, weight, height, and the presence of other chronic illnesses.

### Statistical methods

#### Sample size and power analysis

Power calculations are based on the clinical outcome of BP control using our prior work implementing the ALTA model in a community-based clinic. In the former trial, we found a significant improvement in attainment of BP control at 6 months among patients randomized to the intervention group compared to the UC group (51% vs. 29%, *p* = 0.03). We expect a similar group difference of a 20% increase in BP control between the implementation and UC phases in the current study. Calculations of achieved power were estimated with a stepped-wedge design using Power Analysis and Sample Size (PASS) software program [55]. The power calculations show that we can recruit 10 sites and 700 patients and have at least 80% power to detect a more conservative 15% difference in BP control between the UC and implementation phases for intraclass correlation coefficients (ICCs) ranging from 0.01 to 0.05. Using our original estimate of a 20% difference in BP control, we would have over 90% power to detect a difference between the UC and implementation phases with 10 sites and 700 patients.

#### Descriptive analysis

Baseline characteristics and outcomes will be summarized descriptively and graphically; we will summarize continuous variables with means, standard deviations, medians, and ranges, and will summarize categorical variables with frequency distributions.

#### Analysis of the primary aim

*Qualitative analysis of implementation fidelity* will comprise narrative reports (to assess program adherence), audiotaped health coach sessions (to assess implementation quality), and semi-structured interviews with practice staff/providers and patients (to assess participant responsiveness). Transcriptions of the narrative reports and interviews will be coded using Dedoose software designed for qualitative coding [56]. The coding scheme will be developed by the study team and Clinic Hypertension Committee to focus on key dimensions identified both a priori (i.e., from the interview protocols) and those that emerge during site visits and interviews. Two coders will independently code at least 10 transcripts, after which we will establish the inter-rater reliability [57]. If it is inadequate (Krippendorff’s alpha < 0.80), the study team will work collaboratively to refine and/or clarify the coding scheme and provide additional coder training. Double coding will continue until adequate inter-rater reliability is achieved. Coding the data will allow us to fully describe the themes and the prevalence of specific themes and sub-themes [46].

*Quantitative analysis of implementation fidelity* will consist of descriptive statistics (i.e., mean, standard deviations, frequencies) to provide documentation and descriptions of the practices, implementation components, and context. To describe levels of fidelity to ALTA, each domain of fidelity (adherence, dose, quality, responsiveness, and differentiation) will be coded as described above (i.e., scores will range from 1 to 3). To analyze overall implementation fidelity, we will re-classify each domain into low (score of 0), medium (score of 1), and high levels (score of 2) of fidelity [58]. Overall implementation fidelity will be defined as the sum of all domains and range from 0 to 10. To compare the level of implementation fidelity across the practices, we will use a least square means ANOVA model with weighting by the number of patients seen in each practice within the specified period.

Following the NIH Best Practices for mixed methods research [59], we will construct a joint display [60] that integrates the qualitative themes with the quantitative data to create a composite measure of implementation fidelity based on the five dimensions outlined above. Should the data be divergent, we will assign higher credence to the qualitative data because it provides a richer explanation about participants’ behaviors [59].

#### Analysis of the secondary aims

##### The effect of PF on BP control

Our main clinical outcome is the proportion of patients with adequate BP control (< 130/80 mmHg) at 12 months in the implementation vs. UC phase. BP control will be treated as a dichotomous outcome variable in this analysis; any patients with missing follow-up BP data will be assumed to have inadequate BP control. To examine the difference in BP control rates between the implementation and UC phases, we will utilize a generalized linear mixed model (GLMM) to assess the PF effect. A Poisson regression model will be used to predict BP control specifying a fixed effect for time, a fixed effect for treatment, and a time by treatment interaction. Random effects will be specified for person and practice to account for the clustered nature of the dataset (observations within person and people within practices). In exploratory analyses, will examine the intervention effect on BP reduction at 12 months, treated as a continuous variable. An additional sensitivity analysis will be conducted using GLMM to evaluate the effect of PF on BP reduction. We will test the treatment X time interactions in a random effects linear regression model to determine the time-specific differences BP reduction attributable to PF at the end of the implementation phase.

Randomization of the stepped-wedge design should obviate the need for adjustment, but in the case of imbalanced in baseline covariates, these will be included as necessary in adjusted analyses. All tests will be two-sided with alpha = 0.05 for comparison between the implementation and UC phases. Maximum likelihood estimation of mixed-effects models will be used to account for missing data; in the context of a mixed-effects model, this is equivalent to an assumption of data that are missing at random; we will conduct sensitivity analyses assessing this assumption. We will also compare participants with and without missing values with respect to baseline and practice characteristics. If differential patterns emerge, we will consider the use of multiple imputation and/or inverse probability weighting to adjust for missing data. Analyses will be conducted using Stata, SAS, and R.

Associations between rates of BP control and implementation fidelity will be evaluated using structural equation modeling methods. We will estimate a path model using maximum likelihood estimation to investigate relationships between BP control and implementation fidelity. Implementation fidelity will be identified as individual and simultaneous predictors of BP control. Practice-, staff-, and patient-level variables, which may influence the level of implementation fidelity, will also be included in the model. In addition to the direct effects of each variable, the indirect effects from each variable to BP control via mediator variables will be estimated as the product of component direct effects and tested using bootstrapped 95% confidence intervals. Structural equation modeling analyses will be conducted using R and fit indices will be evaluated to ensure model fit.

##### The effect of PF on medication adherence

We will estimate similar models as BP control with PDC, calculated from available pharmacy records, as the outcome. A GLMM model using maximum likelihood estimation will be utilized to estimate the effect of PF on medication adherence with fixed effects specified for treatment phase, time, and the treatment by time interaction. We will apply any necessary transformations to the PDC outcome to improve the approximation to normality and explore alternative regression strategies, such as rank regression and/or beta regression.

##### The effect of practice and individual-level moderators on implementation fidelity of ALTA

Using the surveys described above, we will explore the potential practice-, provider/staff-, and patient-level moderators that may influence the association between PF and implementation fidelity of ALTA. Potential moderators will be evaluated using structural equation modeling methods and fit indices will be evaluated for all models to ensure adequate model fit.

## Discussion

Poorly controlled hypertension is a major contributor to the racial mortality gap in cardiovascular disease between persons from racial and ethnic minority groups and White persons. National data indicate that Latino patients with hypertension are more likely to have uncontrolled BP (72.4%) compared to White persons (54.4%), despite a similar prevalence of hypertension in both groups (19.5% in White and 17.8% in Latino persons) [61]. Importantly, data from the NHLBI-sponsored Hispanic Community Health Study/Study of Latinos showed significant deficits in the treatment and control of hypertension, with Latino men from Central American, South American, and Dominican ancestry (Latino subgroups largely concentrated in the northeast US) experiencing the lowest rates of BP control (12%, 25%, and 27%, respectively) compared to Mexican American men (31%) [3, 62]. Similar patterns were seen in Latino women. Inadequate blood pressure control due to poor medication adherence may explain the worse outcomes seen in Latino patients.

While research supports the efficacy of multi-level interventions to improve medication adherence in Latino patients [12, 63], there remains a large “translational gap” in implementing these approaches into routine practice in safety-net primary care practice settings. The most common roadblocks to timely translation of evidence-to-practice include trials being conducted in controlled academic clinic settings limiting their external validity, the paucity of purposeful collaborations between academic researchers and practice staff throughout the research process, the failure to assess practices’ needs prior to developing and testing the intervention, and a lack of consideration for the diverse practice contexts [64]. However, as the healthcare system continues to shift toward a value-based payment model, safety-net practices will need to continue to transform their healthcare delivery systems to implement interventions at an accelerated rate.

Our study protocol aims to address this critical need by applying rigorous research methods to identify how efficacious implementation strategies such as PF may work to expedite the translation process for implementing evidence-based practices into safety-net practices to improve health outcomes in Latino patients with hypertension, who suffer disproportionately from poor BP control. Our study will also address critical questions about individual, provider/staff-, and practice-level factors likely to influence the implementation of such approaches. Published reports often omit important details about implementation fidelity, what type of training or assistance practices need, and how these approaches fit within the culture of the practice [16, 65–67]. To our knowledge, this is one of the first studies that assesses implementation fidelity as a multipronged measure to elucidate how best to translate an efficacious intervention into safety-net practices. Previous implementation fidelity research has focused solely on calculating a single fidelity score, determined almost exclusively by measuring adherence to the study protocol [46]. Thus, a large gap remains in our understanding of how and why efficacious interventions may (or may not) be effective when translated to real-world settings. Moreover, data linking variation in clinical outcomes with variation in organizational factors (e.g., adaptive reserve) are often missing. Our use of a rigorous mixed methods approach that examines the five core dimensions of implementation fidelity, as opposed to a singular construct, will allow us to document and address any observed variability in process and clinical outcomes across the practices.

### Limitations

There are several methodological challenges that we may encounter during the course of the study. First, there are limitations to collecting our clinical outcome data through the EHR. Data on medication adherence (PDC metric) are based on prescription refill claims, which may miss instances where patients pay out-of-pocket for medications or where pharmacies fail to render claims for prescriptions they fill. This could result in a PDC that appears lower than true adherence. We estimate that 10% of patients would pay out-of-pocket for their antihypertensive medications [68]. Another potential limitation is that patients may refill their prescriptions but not take the medication, resulting in a high PDC but low adherence. To address these limitations, we will collect information on patients who pay out-of-pocket for their medications and ask patients to report on their level adherence over the past month using a well-validated self-report adherence scale [52, 53]. Another limitation is the potential variability in the measurement of BP by different clinic staff. We will address this by using the mean of the last two readings in the EHR. Finally, changes in the external healthcare context may affect implementation and clinical outcomes (e.g., changes in reimbursement for medications). We will collect data on changes to the external healthcare context throughout the study to account for any threats to internal validity in our analyses.

### Implications

The goal of ALTA is to test PF as a strategy for accelerating the implementation of an evidence-based multi-level intervention for improved hypertension management into routine practice within “real world” safety-net primary care settings. To facilitate sustainability, ALTA is designed to complement other quality improvement initiatives currently underway at the practices such as Meaningful use, Patient-Centered Medical Home, and attaining Healthcare Effectiveness Data and Information Set [69] quality metrics, including BP control as well as supporting practices’ efforts to receive Nursing MAGNET status.

ALTA combines a rigorous research framework with a mixed method approach to evaluate the effect of a tailored PF strategy on the implementation process (fidelity) and clinical-effectiveness (BP control, medication adherence) of a multilevel approach to improving hypertension control in safety-net practices. By examining the barriers and facilitators that affect the implementation of ALTA at each level, our study will add knowledge that will increase the generalizability of our findings to other safety-net practices and guide effective scale-up across primary care practices nationally. Moreover, the knowledge gained from this study will provide key academic, community, and policy stakeholders a practical and replicable strategy for implementing efficacious interventions into routine care that has potential for broad dissemination, ultimately contributing to the reduction of health disparities seen in racial and ethnic minority populations.

## Data Availability

Not applicable

## Abbreviations

AHRQ: Agency for Healthcare Research and Quality
ALTA: Advancing Medication Adherence for Latinos with Hypertension through a Team-based Care Approach
BP: Blood pressure
CFIR: Consolidated Framework for Implementation Research
EBP: Evidence-based practice
ECHO: Experience of Care and Health Outcomes
EHR: Electronic health record
GLMM: Generalized linear mixed model
HEDIS: Healthcare Effectiveness Data and Information Set
HIT: Health Information Technology
ICC: Intraclass correlation coefficient
IOF: Implementation Outcome Framework
MAs: Medical Assistants
NYULH: New York University Langone Health
PASS: Power Analysis and Sample Size
PDC: Proportion of days covered
PF: Practice facilitation
UC: Usual care
US: United States
