# Correction to: Bridging the evidence-to-practice gap: a stepped-wedge cluster randomized controlled trial evaluating practice facilitation as a strategy to accelerate translation of a multi-level adherence intervention into safety net practices

**DOI:** 10.1186/s43058-021-00226-6

**Published:** 2021-10-21

**Authors:** Antoinette Schoenthaler, Franzenith De La Calle, Amanda Soto, Derrel Barrett, Jocelyn Cruz, Leydi Payano, Marina Rosado, Samrachana Adhikari, Gbenga Ogedegbe, Milagros Rosal

**Affiliations:** 1 Department of Population Health, Center for Healthful Behavior Change, NYU Langone Health, 180 Madison Avenue, 752, New York, NY 10016 USA; 2 Preventive and Behavioral Medicine, Department of Medicine, University of Massachusetts Medical School, Worcester, MA USA

**Correction to: Implement Sci Commun 2, 21 (2021)**

**https://doi.org/10.1186/s43058-021-00111-2**

Following publication of the original article [1], it was reported that the definition of blood pressure (BP) control for the secondary outcome was incorrectly stated as systolic BP < 130 and diastolic BP < 90 mmHg. The correct definition of BP control is systolic BP <140 and diastolic BP <90 mmHg.

The original article has been corrected.
